# Legal and ethical reflections on the use of artificial intelligence in the diagnosis and treatment of cancer: who assumes responsibility?

**DOI:** 10.3389/frai.2026.1812408

**Published:** 2026-04-15

**Authors:** Virgiliu-Mihail Prunoiu, Ovidiu Juverdeanu, Codruta Cosma, Simion Laurentiu, Victor Strâmbu, Adrian Radu Petru, Mihai Stana, Mircea-Nicolae Brătucu

**Affiliations:** 1 “Carol Davila” University of Medicine and Pharmacy, Bucharest, Romania; 2 Clinic I General and Oncological Surgery, “Prof. Dr. Alexandru Trestioreanu”, Oncological Institute, Bucharest, Romania; 3 Law Offices Ovidiu Juverdeanu, Bucharest, Romania; 4 Department of Surgery, General Surgery Clinic, Clinical Hospital “Dr. Carol Davila”, Calea Griviței, Bucharest, Romania; 5 Department of General Surgery I, Emergency Clinical Hospital Bucharest, Calea Floreasca, Bucharest, Romania

**Keywords:** artificial intelligence (AI), cancer diagnosis, diagnostic algorithm, legal regulation, machine learning, malpractice, robotic surgery

## Abstract

Artificial intelligence (AI) offers multiple advantages, such as: improvement and accuracy of the diagnosis, decrease of the doctors’ workload, decrease of the hospitalization costs, and becoming increasingly widespread, studied, and applied in medicine. AI is already used in image recognition, has haptic perception, and can manipulate instruments. Thus, surgical robots will likely be driven by AI. In the near future, machine learning (ML) will also appear. The use of AI and the study of the specialty literature raise ethical and legal questions for which there is no unanimous answer yet. Medical liability (malpractice) for AI-related errors and damages to the patient prompts legal reflections on this topic. The diagnostic algorithms of AI raise questions regarding the risks of using AI in the diagnosis and treatment of cancer (especially in rare cases), the information provided to the patient, all of these having moral and legal implications, as well as regarding the impact on the empathic doctor–patient relationship. Actually, the use of AI in the medical field has triggered a revolution in the doctor-patient relationship, but it has possible medico-legal consequences as well. The current legal framework regulating medical liability when AI is applied is inadequate and requires urgent measures, because there is no specific and uniform legislation to regulate the liability of the various parties involved in applying AI, or that of the end-users. Consequently, greater attention should be paid to the risk of applying AI, to the necessity to regulate its safe use, and to maintain the safety standards of the patient by continuously adapting and updating the system.

## Introduction

In contemporary medical practice, the application of artificial intelligence (AI) algorithms represents a new situation that helps doctors, both during the diagnostic stage and during treatment, who are increasingly being used in hospitals. AI enables healthcare professionals to obtain more accurate and more precise diagnoses, as well as to provide more efficient and less invasive medical and surgical treatments. At present, AI is used successfully for the imaging diagnosis of tumors, as well as in oncology, for its potential to recognize complex patterns which provide quantitative and qualitative assessments, improving the prediction of the diagnosis and treatment (Cestonaro et al., 2023). Through risk-stratification models, AI may optimize the resource allocation plan, proving useful in-patient management, research, and clinical trials (Cestonaro et al., 2023). However, although AI systems are increasingly used in medicine, the ethical implications and the legal regulations regarding their use remain under debate. The human–machine interaction needs to be analyzed, particularly when AI systems make autonomous choices, which raise legal liability issues in case the patients are harmed. The new technological approaches create a new reality which is unlikely to fit within the limits of the current legislation. In this context, this paper aims to address both the critical aspects of using AI algorithms in the diagnosis and treatment of cancer and to discuss the legal liability associated with the use of the method and the potential solutions (Cestonaro et al., 2023).

## Questions and answers

The abundance of information and published articles (PubMed, social media, Google, etc.) at this time related to the application of AI in medicine led me to opt for a more “sensitive” field, namely, the application of AI in oncology. I believe that oncology patients can benefit more from using the system both for rapid diagnosis (including for screening) and for the application of personalized treatment and the development of new molecules (the Nobel Prize in Chemistry in 2024). But, within the treatment, I chose the surgical one, where I believe that human-machine interaction is important for the development of future surgery, and especially robotics.

Related to these aspects, through this article, we aimed to study the specialized literature so that the doctor-patient-AI system interaction is also seen from a legal point of view (malpractice—sharing of responsibility between clinicians, hospitals and providers), by updating European legislation in the field, presenting legal and ethical reflections and proposing guidelines that do not hinder the development of AI systems.

### AI can revolutionize medicine!

Thus, they can provide predictions on the evolution and prognosis of a type of cancer following systemic chemotherapy treatment and surgical treatment, recommended following presentation of the case to the specialized oncology committee (renal, prostate, breast cancer, etc.). Some parameters are constantly generated by the healthcare system: blood pressure values, laboratory test results, oxygen saturation, and results from ultrasound, mammography, CT—Computed Tomography, MRI—Magnetic Resonance Imaging, or PET-CT—Positron Emission Tomography and Computed Tomography examinations. The information is evaluated and used for diagnosis and archiving (AI in medicine, 2026). Radiomics also comes into play here, a related field of AI that involves the extraction of imaging data about a tumor (shape, size, density, etc.). These are correlated with medical images processed using advanced mathematical algorithms to discover tumor characteristics that cannot be appreciated with the naked eye. Radiomics has great potential to provide important phenotypic information, such as intra-tumor heterogeneity, its appearance, staging, and this helps the doctor in making a personalized therapeutic decision, but also in assessing the prognosis of tumor evolution (lung cancer, breast neoplasm, etc.). Radiomic characteristics offer an objective and quantitative way to evaluate the tumor phenotype, having a wide range of possible applications in oncology by differentiating benign and malignant tumors (AI in medicine, 2026; Yip and Aerts, 2016).^1^

### Can a computer learn “in a controlled manner”?

AI represents the capacity of computer programs to learn. There are two possibilities for learning. The first is “supervised learning,” in which a large number of similar things are presented to the computer, for example, a large number of imaging investigations, thereby teaching it which are normal or suspected of neoplasm, based on the images provided and, on their description, including the comparison and association of data obtained through histopathological and immunohistochemical (IHC) examination. If the computer receives sufficient information, it will be able to distinguish between the images provided. Thus, it can not only make the work of the doctors easier, but it can also improve the quality of the diagnosis and of the personalized treatment (AI in medicine, 2026). AI can thus lead to a decrease in the time spent on diagnosis and an increase in the capacity to examine a greater number of patients. For example, for breast cancer, AI can help with the fast diagnosis of the disease, its staging, the choice of a personalized treatment, and the assessment of the prognosis with the establishment of the disease-free interval (Cestonaro et al., 2023). AI technologies should be considered complementary to the diagnosis, enhancing the performance of the doctors, reducing the risk of human error, and serving as clinical decision support. Furthermore, AI can filter simple cases and allow specialists to focus more on the more difficult cases. The advantages could also include its use in cancer screening (breast, cervical, colorectal, etc.) and the early identification of individuals at increased risk of developing this disease (Cestonaro et al., 2023; AI in medicine, 2026; Yip and Aerts, 2016; Tripathi et al., 2024; AI Predicts Future Pancreatic Cancer, 2025; Terranova et al., 2024; see Footnote 1). These applications are tools that help with the diagnosis, but they do not replace the clinicians. In addition to clinical diagnosis, AI may assist surgeons intraoperatively, leading to more precise, minimally invasive surgical techniques (Cestonaro et al., 2023). Furthermore, when AI begins to function autonomously (machine learning—ML), without specialists, based on a self-learning algorithm (“the second stage of learning”), it may make a diagnosis without the necessity of the clinician’s approval.

*Then, should the system not be held responsible for incorrect results, and should the AI programmers or the software-selling company not assume at least partial medical liability?*

*Is the medical clinical examination still useful?* This also raises questions concerning the legal liability in the case of diagnostic errors due to the absence of a physical examination. *Will AI and machine learning (ML) ever manage to replace “*a skilled, empathic clinician at the bedside of the patient”? Yes, would be the answer now and in the future, because AI would do the same thing repeatedly, i.e., see patients, make a diagnosis, and propose treatment options, without becoming tired and without losing patience, for a large number of patients. *Will young doctors still want to specialize in the fields where AI can make the diagnosis on its own (radiology)? And in that case, how is medical fault established? What about malpractice?*

## AI in the diagnosis and treatment of cancer (clinical decision support systems—CDS)—huge potential, huge risks

Multiple political and legislative options could ensure a more balanced liability system, including adjusting the standard of care, medical insurance, compensation, and legal regulations. In practice, with an adequate legislative framework, the doctors could facilitate the safe and speedy implementation of AI, as well as its autonomous learning (machine learning—ML), ensuring modern and efficient care for the patients (Parlamentul European, n.d.; Hotărârea Guvernului nr, n.d.; Maliha et al., 2021).^2^ In the USA (2017), the FDA (Food and Drug Administration) approved the use of AI in medicine (X-rays, biopsies—prostate cancer, etc.; Ström et al., 2020), but the question remains as to who is liable for the incidents/inconveniences caused to the patients. The solution will depend on how much innovation in AI/ML is appropriate, and its balanced introduction into the healthcare system will also have to take into account compensation for the injured patients. The level of liability directly influences the level of development and implementation of the clinical algorithms. Increasing liability may discourage doctors, healthcare systems, and AI designers, hindering or delaying the development and implementation of these algorithms. Likewise, research in this field is discouraged by increasing costs (Maliha et al., 2021). The AI/ML systems are improving and advancing at a rapid pace, whereas the legal system moves more slowly and has to adapt and balance progress with liability to promote innovation, safety, and the adoption of these new methods of diagnosis and treatment (Maliha et al., 2021).

From an ethical point of view, the question arises whether the application of AI/ML still meets the medical standards of the Hippocratic Oath (Naik et al., 2022). Nonetheless, science is advancing, and systems such as the Artificial Intelligence System (AIS) from IBM Watson—a program for oncology (IBM Watson for Oncology SaaS—5725-W51)—have the mission to support oncologists, thus directly influencing clinical decision-making. With over 30 billion images available for analysis, AIS could evaluate the information provided for the diagnosis of neoplastic disease and recommend personalized treatment for the patient. The most recent systems have a broad coverage which includes breast, lung, colorectal, gastric, and cervical cancer (IBM, 2024; Zou et al., 2020; Jie et al., 2021; Printz, 2017; Shah et al., 2020; Griffin, 2021). The recommendations for the diagnosis and treatment of cervical cancer using WFO (Watson for Oncology) were consistent with real-world clinical practice in 72.8% of cases. Factors such as the location of the cancer, as well as individual factors such as the type of chemotherapy used, the surgical procedure chosen, and the adjuvant/neoadjuvant therapy, limit its wider application. The conclusion was that WFO cannot replace oncologists (the tumor board) in supporting the diagnosis and recommending personalized treatment for patients with cervical cancer, but it might be an efficient tool in decision-making and therapy standardization (Zou et al., 2020). Regarding the concordance between the diagnosis and the treatment established using WFO, it was the highest for breast cancer (88.99%) and the lowest for gastric cancer (57.94%), whereas the concordance rate was higher in stages I-III than in stage IV (Jie et al., 2021). It should be noted that while it takes a tumor board between 12 and 20 min to make a diagnosis and recommend personalized treatment, it takes the WFO system 40 s to do the same, and it can offer consultations to an unlimited number of patients (Printz, 2017). Some authors consider that in the near future, AI should be included in the diagnosis and treatment guidelines (renal cancer, prostate cancer, etc.), thus revolutionizing decision-making (Shah et al., 2020). It is estimated that by 2030, AI will influence medical practice by 14%, intervening in the analysis of electronic medical records, processing laboratory data, and analyzing medical imaging, providing diagnoses and therapeutic solutions, all done quickly and with a high cost/efficiency ratio. The data obtained may be used both in medical research and in the development of new drugs (The Nobel Prize in Chemistry 2024—David Baker, Demis Hassabis, John Jumper; Griffin, 2021; Bradford et al., 2017; The Nobel Prize in Chemistry, 2024).

Doctors will have to inform the patients about the benefits, risks, and limitations of using AI and ask for their consent. The critical issues related to the use of AI in medicine are confidentiality and cybersecurity: the effectiveness of the software relies on the use of numerous data points, and some of them may have an impact on the private lives of the patients (Cestonaro et al., 2023).

## AI and robotic surgery

As far as surgical interventions are concerned, according to predictions, robots equipped with artificial intelligence could replace doctors in treating patients in the near future (Griffin, 2021). The use of AI may be divided into 2 branches: a component with a material application, such as surgical robots, and a virtual component—the patient’s electronic file—with the establishment of the diagnosis and of the treatment (*clinical decision support systems,* CDS; Griffin, 2021; Bradford et al., 2017). The undeniable advantages of robotic surgery, successfully applied in oncological surgery as well, are well known, such as: high precision and increased control of the intervention, improved visualization-3D, reduced intraoperative blood loss, fewer postoperative complications, faster patient recovery, and shorter hospital stay.

Robots guide the surgeon in the three-dimensional space by tracking the movements of the instruments during the surgical procedure, using optical or electromagnetic sensors. The computer and the robot alert the surgeon about the precise location and spatial orientation of the surgical instruments, providing feedback on the danger to surrounding anatomical structures and the placement and orientation of the surgical instruments. Many robots have “haptic” sensors, which provide increased resistance to tissue movement to provide the surgeon with feedback during the surgery. Thus, robots define the haptic boundaries for the surgical instruments and provide haptic feedback when the surgeon deviates from the safety area created by the preoperative surgical planning in the form of tactile or auditory and visual alerts, preventing injury to the organs outside the operating field (Griffin, 2021). Today’s robots are semi-automated, helping the surgeon to perform extensive minimally invasive surgical maneuvers in three-dimensional space by positioning the instruments, but having a “passive” attitude, although theoretically the robots could perform certain maneuvers. These are successfully used in colorectal cancer surgery, gynecological oncology, prostate and renal cancer, lung cancer, etc. (Griffin, 2021; Bradford et al., 2017; The Nobel Prize in Chemistry, 2024; Pandav et al., 2022).

## Machine learning

Machine Learning (ML) enables computers “to receive data and learn on their own” from “examples rather than from a list of instructions”. ML uses methods that gather algorithms to mimic human thinking, “enabling computers to make predictions by using substantial quantities of patient data, by learning their own associations”. By using substantial quantities of patient data, “machine learning can create algorithms which function similarly to the reasoning of doctors”. ML can also learn and “take into account variables and interactions, make often unexpected predictions, and offer solutions not previously described in the literature and previously unknown to researchers”. It can thus make predictions about the evolution of the disease of an oncological patient, about its prognosis, and offer therapeutic solutions. It can revolutionize healthcare by enabling doctors to identify “which patients may develop certain diseases”, “which patients should be examined more often”, which patients should be treated more aggressively”, and to select the most appropriate specific treatments (personalized medicine; Bradford et al., 2017; The Nobel Prize in Chemistry, 2024; Pandav et al., 2022; Parikh et al., 2019).

Deep learning is the most advanced part of ML. It uses advanced computational models which “allow an algorithm to program itself by learning from a large set of examples, removing the need to specify the rules explicitly”. In practice, the computer acts autonomously in decision-making. Deep learning has been used by companies such as Google and Facebook to analyze “big data” to “anticipate what people search for on the internet: where they wish to travel, what they like to buy, what they prefer to eat, and what kind of friends they make” (Bradford et al., 2017; The Nobel Prize in Chemistry, 2024; Pandav et al., 2022; Parikh et al., 2019; Wong and Bressler, 2016). Medical applications include imaging, diagnosis, and establishing the treatment for a given disease. An example is the prediction in the diagnosis and treatment of skin cancer (melanoma and non-melanoma tumors) using the computerized analysis of photographic images and the gene expression profile (GEP) obtained through biopsy (Wei et al., 2024). Similarly, for breast cancer, the analysis of the anatomopathological specimens is fast, and the diagnosis is comparable to that made by the best pathologist (Zhou et al., 2020). AI can analyze the CT scans of a patient with cancer and, by combining these data and by learning from previous patients, it can recommend patient-tailored radiotherapy which aims to minimize injury to adjacent organs. The work of radiation oncologists is made easier by reducing the time for treatment planning, quality control, and standardization (Zadnorouzi and Abtahi, 2024; Kawamura et al., 2024).

Liability for AI errors is an important issue, and even though numerous papers have been published, there is currently no unanimous and definitive answer to this issue. AI is a new technology, and the legal sanctions applicable to it are not yet well-developed. In the case of a malpractice lawsuit, the question is whether there was a deviation from the standard of care for the patient. In this context, AI may fail in the use of programming algorithms and in guiding doctors (Terranova et al., 2024). The following dilemma arises: if the manufacturer is liable for a medical device in case of malfunction and harm to the patient, in the case of AI use, is the doctor, who approves the AI results, liable for them? They must inform the patients about the limits of using the system, and the responsibility of the clinicians should be narrowed. In practice, if a robotic surgical procedure is performed autonomously, the injuries caused by software configuration defects would likely be the responsibility of the developer. At the same time, programmers should not be held fully liable, since AI is not capable of preventing injuries in all cases. The legislation will have to be based on the degree of autonomy of the software, and when AI is used only as a decision support in an activity, the doctor who approves it bears the risk of liability, even if it is “indirect” (Cestonaro et al., 2023; Terranova et al., 2024).

AI products offered to doctors that have a defect for which the manufacturer is responsible could be an obstacle to the development of the initial algorithm, although it may improve in time. This is why the information provided to the patients about the risks, benefits, and limitations of AI use is of paramount importance and should aim to ensure they are fully informed and make conscious choices. All of these represent informed consent, which is the legal foundation of the doctor’s duty to inform the patient. The topic of consent is essential given the application of personalized treatment, including the use of AI in medicine, which must ensure confidentiality, without discriminating against populations based on ethnicity and gender, and guarantee equal, fair access to resource allocation.

## Liability for the use of AI in medicine—principles

### Legal liability

Several questions arise: *Who is legally liable for the use of AI in autonomously operating systems in the medical field?* Does the same law apply to cars that drive/park autonomously or to airplanes flying on autopilot? *Who is liable when a surgical robot using AI has a manufacturing defect?*

Design defect—The specialty literature shows that there are few cases in which surgical robots have design defects. Bearing in mind that robotic surgeries have increased by 250% in recent years, there have been few malpractice lawsuits on this topic. The specialties with the highest number of complaints were obstetrics/gynecology (48.9%), general surgery (28.9%), and urology (15.6%; De Ravin et al., 2023). Therefore, the person making the malpractice claim should distinguish between the design defect of a robot using AI and the action of the doctor who performed the surgery. This is why we believe that doctors must supervise the application of AI in robotic surgery, and the VR system (virtual reality) could help surgeons to learn faster how to work with robots (Bradford et al., 2017; Riddle et al., 2024).

Estimated risks—the application of a new product also entails usage risks that may be attributable to the manufacturers. The use of AI includes predictable risks common to many medical devices, such as malfunction, improper use, and wear and tear due to the use of the devices. All of these should involve legal liability similar to the liability for non-AI products. Some of the predictable risks are unique and associated with AI, such as erroneous data input, faulty algorithm application, discrimination, IT- system failures, etc. (Bradford et al., 2017).

Erroneous information (Bad Data)—AI and deep learning depend on the quality of the data supplied (Verghese et al., 2018). Billions of medical data points are used to generate models, and if these are not monitored, they can amplify errors. Therefore, it is essential to ensure clarity and comprehension when introducing algorithms, and the data must be reproducible. There are several factors which influence this activity and can generate errors: the consistent volume of information, its quality, the scope and complexity of the information from the medical field (especially in oncology), its distribution and development over time (constantly changing medical guidelines due to the emergence of new methods of diagnosis and treatment), and last but not least, the interpretation of this information. Regarding the volume and quality of the data, although the number of patients/day that an AI system can “see” may be limited, the volume of processed information is huge. At least 10 parameters are necessary to make a correct diagnosis (Derek and Grant, 2019; Ibrahim et al., 2024). These data are far more complex than voice and image recognition (Bradford et al., 2017). At the same time, care must be taken that, if the system is used for the prognosis of a neoplastic disease, and the data analysis shows a life expectancy of less than 6–12 months, the patient should not remain without medication (Bradford et al., 2017).

It is obvious that the accelerated application of AI in medicine will lead to claims/complaints regarding medical negligence and that, for this reason, on the one hand, the legal system should provide clear solutions regarding which entity bears liability, but on the other hand, the medical malpractice insurance system should state the terms of coverage in the cases where healthcare decisions are partially/fully made by AI.

### Malpractice

By using AI in medicine, the system brings new elements to the complexity of malpractice, especially since the legislation in this domain is in its infancy. Doctors must ensure a human interface to AI, carefully interpreting the data provided by the system and making correct clinical decisions. Doctors devote an average of 15 min to a patient’s history, but with AI, they could see many more patients. This means that doctors are “obliged to have a standard of learning and skills”—continuous medical education. From this, it can be deduced that, sooner rather than later, with the introduction of AI into the medical practice of diagnosis and treatment, “doctors must know how the law will hold them liable for the physical and psycho-emotional injuries resulting from the interaction between algorithms and patients” (Bradford et al., 2017). AI will rapidly influence the diagnosis and treatment of patients, predicting who will develop a certain disease and recommending the appropriate treatment, and it may become part of “the standard of care and efficiency in treating patients”. At present, in order to avoid the absence of adequate legislation in the field, doctors prefer to use AI as a tool to confirm their work rather than as a source of improvement (Bradford et al., 2017; Ibrahim et al., 2024). These aspects will have to be included in a new malpractice law that protects the doctors who use AI; otherwise, they will have to appeal to the courts to defend their decisions. Therefore, there is a risk that doctors will avoid applying AI. For example, a surgeon who uses an AI-assisted robot risks being subject to malpractice if this procedure is not covered by the rules of use for the respective surgery, and will avoid applying this technique. Thus, during a robotic operation performed with AI, the surgeon will only have to supervise certain surgical times, but he/she will have to correct any possible problems that arise at the end of the operation (Bradford et al., 2017). This shows that surgeons must also master classic/laparoscopic surgical techniques.

At this time, it is difficult to prove whether the patient’s injury was caused strictly by the manufacturer of the AI-equipped robot or by the faulty use of the system by the surgeon. It is also possible that the hospital that owns the robot and is responsible for its maintenance is to blame. Thus, the following question arises: in such a case, who does the malpractice law sanction? This is an aspect to which jurists must adapt the law. It must be taken into account that AI and ML can optimize an oncological treatment (Sagiv, 2025) and can consult an immense number of patients/days, which would reduce the time for scheduling them for personalized diagnosis and treatment, while also providing a prognosis of the evolution of the disease. It is inevitable that with the increase in the application of AI, the number of malpractice cases will also increase. It should not be forgotten that the patient must be informed about the use of the AI system and consent to it.

## Discussion

A lot of personal information circulates in the media, but when discussing access to medical data to be used for AI research and development, numerous obstacles must be overcome. GDPR—General Data Protection Regulation is extremely important; however, considering only this, progress in AI will not be possible. Practically speaking, it is not the names and personal data that matter; it is the medical information obtained from the patients that is important, and it is difficult to collect medical data for research if it is not available! Nevertheless, in many countries around the world, politicians and legal experts are becoming involved in solving this problem, as there is a true will for the advancement of AI (Parlamentul European, n.d.; Hotărârea Guvernului nr, n.d.; see Footnotes 1, 2).

### Legal point of view

We present below the current legal regulations regarding the use of AI in the diagnosis and treatment of cancer. The European Parliament (EP) has adopted “The Artificial Intelligence Act”, which guarantees the safety and respect for the fundamental human rights and stimulates innovation. The law aims “to *protect fundamental rights, democracy, the rule of law and environmental sustainability from high-risk AI systems, to encourage innovation and to ensure a leading role in the field for Europe*” (Parlamentul European, n.d.; see Footnote 2). The regulation imposes obligations concerning the use of AI according to its potential risks and the anticipated impact. The law responds to the proposals for “a safe and trustworthy society, including by fighting against disinformation and ensuring that people ultimately have control, ensuring human oversight, as well as reliable and responsible use of AI”. The framework convention will be open for international ratification and enables countries around the world to adhere and to comply with the established ethical and legal norms, in line with the vision of the Resolution of the General Assembly of the UN of 21 March 2024 (Parlamentul European, n.d.; see Footnote 2). The Romanian Government has also adhered to this legislation by publishing Government Decision no. 832/2024 in the Official Gazette of 25.07.2024 regarding the approval of “The National Strategy in the Field of Artificial Intelligence” (Hotărârea Guvernului nr, n.d.).

One of the critical points of the legal regulations consists of the difficulty of adapting to the new realities. We consider that a regulation is considered evolved to the extent it manages to capture within itself its own events, which are different from typical norms, it anticipates future events, and which can be subsumed by it: this can be achieved only if it includes broad definitions and, at the same time, clear, precise, and comprehensive recommendations.

Medicine is not a science guided solely by statistics, mathematics, and algorithms. The question is how AI can interact with human feelings as an important part of the art of medicine, such as “touch, compassion, and empathy”? Illness in general and the diagnosis of cancer in particular represent more than a simple physical examination or a laboratory test, and from this point of view, the doctor-patient relationship is important. From an ethical point of view, communication between doctor and patient, and between doctor and family, is important. “Today’s technology is not yet sensitive enough to capture nuanced social signals, such as body language, tone of voice, and the emotional state at the respective moment.” (Bradford et al., 2017; The Nobel Prize in Chemistry, 2024; Pandav et al., 2022; Parikh et al., 2019; Wong and Bressler, 2016; Wei et al., 2024; Zhou et al., 2020; Zadnorouzi and Abtahi, 2024; Kawamura et al., 2024; De Ravin et al., 2023; Riddle et al., 2024; Verghese et al., 2018; Derek and Grant, 2019).

It is important when the algorithm for AI and the use of electronic records is created, because there are cases in the literature where complaints have appeared regarding the method of making the diagnosis and the prognosis of the disease’s evolution. The legal system should consider the following questions: *Did AI perform as well as a person? Are the performances of the AI system in accordance with those described by the manufacturer? Is the legislation prepared to answer such questions?* (Bradford et al., 2017).

In the following paragraphs, we will present what legislation we rely on today and how the evolution of AI is seen from a legal point of view.

A) *The current legal framework*

Liability for malpractice when using artificial intelligence (AI) in medicine is a developing field, with regulations varying according to the jurisdiction. In the European Union (EU) and in Romania, the current regulations regarding medical liability apply to AI as well, but legislative initiatives to adapt them are underway.

1. *The general framework applicable in Romania and the EU*

- *The Civil Code and Law no. 95/2006 regarding the healthcare reform* regulate the civil liability of medical professionals. The doctor, the medical facility, or the manufacturer of a medical device may be held liable if the use of AI causes harm. (Law no. 95/2006 on the reform in the field of healthcare, n.d.; Civil Code, n.d.)
- *Regulation (EU) 2017/745 regarding medical devices (MDR—Medical Devices Regulation)—Regulation EU 2017/745 Of The European Parliament and of The Council of 5 April 2017 on medical devices, amending Directive 2001/83/EC, Regulation (EC) No 178/2002 and Regulation (EC) No 1223/2009 and repealing Council Directives 90/385/EEC and 93/42/EEC)—*sets strict requirements for AI-based medical software, particularly in terms of safety and performance [Regulation (EU) 2017/745 on medical devices (MDR), n.d.].
- *The GDPR (General Data Protection Regulation)* imposes requirements regarding the protection of medical data, particularly in the case of AI algorithms that process sensitive information. *(Regulation EU) 2016/679 of the European Parliament and of the Council of 27 April 2016 on the protection of natural persons about the processing of personal data and on the free movement of such data, and repealing Directive 95/46/EC* [*General Data Protection Regulation;* Government of Romania, 2000; European Parliament, for EU laws, 2016].

2. *Who is liable in case of harm caused by AI?*

- *The doctor*, if he/she uses AI as a diagnostic tool and make a wrong decision based on its recommendation. Although AI may support the medical decision, the final responsibility lies with the doctor.
- *The provider of medical services*, such as a hospital or clinic, uses a faulty AI system and fails to implement adequate safety measures.
- *The AI software manufacturer* may be liable if the algorithm has systemic errors, offers faulty recommendations, or does not comply with the MDR standards.

3. *Current and Future Regulations*

- *The AI Act (the EU Regulation on Artificial Intelligence, proposal) Artificial intelligence (AI) act: Council gives final green light to the first worldwide rules on AI [Regulation laying down harmonized rules on artificial intelligence (artificial intelligence act), 21 May 2024]—*will impose stricter requirements for the AI systems used in medicine, classifying them as “high risk” and will oblige manufacturers to ensure transparency, safety, and the possibility of human oversight [Regulation laying down harmonised rules on artificial intelligence (artificial intelligence act), 2024]
- The Directive on Liability for Defective Products (revised in 2024) extends the liability of the manufacturers to software, including medical AI. *Directive (EU) 2024/2853 of the European Parliament and of the Council of 23 October 2024 on liability for defective products* (Directive (EU) 2024/2853 of the European Parliament and of the council of 23 October 2024 on liability for defective products, 2024)

To conclude, the liability for malpractice in the case of AI in medicine depends on the human factor involved, as well as on the obligations of the manufacturers and of the providers of medical services. In the future, the AI Act will clarify this responsibility even further.

### Liability for malpractice when using artificial intelligence in medicine—detailed legal analysis

The use of artificial intelligence (AI) in the medical field raises complex legal issues regarding liability in the event of harm. In the absence of specific exhaustive regulations, a general legislative framework applies in Romania and the European Union, supplemented by recent initiatives aimed at adapting the norms to the new technological realities.

#### The normative framework applicable in Romania and the European Union

##### Medical liability and malpractice

In Romania, liability for malpractice is regulated by:

- *Law no. 95/2006 regarding the healthcare reform*—The chapter regarding civil liability for medical malpractice (art. 642–689; Law no. 95/2006 on the reform in the field of healthcare, n.d.).
- *The Civil Code*—General principles of tort liability (art. 1,349 and the following; Civil Code, n.d.).
- *Government Ordinance no. 137/2000 regarding the prevention and sanctioning of all forms of discrimination*—Relevant in cases of algorithmic bias in medical AI (Government of Romania, 2000).

These regulations establish the liability of the doctors, healthcare facilities, and other actors involved in providing medical services.

##### The liability of the manufacturers of medical software based on AI

AI used in medicine can be classified as a *medical device* according to:

- *The Regulation (EU) 2017/745 regarding medical devices (MDR; Regulation (EU) 2017/745 of The European Parliament and of The Council of 5 April 2017 on medical devices, amending Directive 2001/83/EC, Regulation (EC) No 178/2002 and Regulation (EC) No 1223/2009 and repealing Council Directives 90/385/EEC and 93/42/EEC),* which classifies medical software as a medical device and imposes strict requirements for safety, performance, and post-market surveillance [Regulation (EU) 2017/745 on medical devices (MDR), n.d.].
- *The Regulation (EU) 2017/746 regarding in vitro diagnostic medical devices (IVDR), (Regulation EU 2017/746 of the European Parliament and of the Council of 5 April 2017 on in vitro diagnostic medical devices-IVDR)—*applicable to AI used in the diagnosis of diseases through laboratory tests [Regulation (EU) 2017/746 of the European Parliament and of the council of 5 April 2017 on in vitro diagnostic medical devices, 2017].
- *EU Regulation No 988/2023 on General Product Safety (GPS) applicable from 13 December 2024 (the “GPS Regulation”)—[Regulation (EU) 2023/988 of the European Parliament and of the Council of 10 May 2023 on general product safety—GPS;* Regulation (EU) 2023/988 of the European Parliament and of the Council of 10 May 2023 on general product Safety, 2023*]*
- For the same reasons that led to the adoption of the *SGP Regulation*, on 18.11.2024, *EU Directive No. 2024/2853 on liability for defective products (LPD) and repealing Directive No. 85/374 (the “LPD Directive”)* was published. The Directive is to apply to products placed on the market or put into service after 9 December 2026, and by 9 December 2026, Member States must ensure the entry into force of the regulatory acts transposing this new Directive [Directive (EU) 2024/2853 of the European Parliament and of the council of 23 October 2024 on liability for defective products, 2024]
- The *LPD Directive* will apply to any product understood as any movable property, even if it is integrated into another movable or immovable property or if it is interconnected with it, and will also include digital production files, as well as software [Directive (EU) 2024/2853 of the European Parliament and of the council of 23 October 2024 on liability for defective products, 2024].
- Software may be placed on the market as a stand-alone product and subsequently integrated into other products as a component, potentially causing damage when operated. Therefore, for reasons of legal certainty, the LPD Directive will clarify that software is a product for strict liability, regardless of how it is supplied or used, and therefore, regardless of whether it is stored on a device or accessed via cloud technologies. However, the source code of software should not be considered a product for this Directive, as it is merely a set of information [Directive (EU) 2024/2853 of the European Parliament and of the council of 23 October 2024 on liability for defective products, 2024].
- Liability for defective products is objective liability, meaning that no fault is required. Liability lies with the manufacturer of the product, the manufacturer of a defective component, and if the product or component is produced outside the EU, then the importer or the manufacturer’s authorized representative may also be held liable. If there is no importer or authorized representative, then the logistics service provider may be held liable. Software developers or manufacturers, including providers of AI systems within the meaning of the (EU) AI Regulation, will be considered manufacturers and treated as such [Directive (EU) 2024/2853 of the European Parliament and of the council of 23 October 2024 on liability for defective products, 2024].

Non-compliance with these regulations may entail the liability of the software manufacturers, distributors, and providers of medical services.

##### Data protection and confidentiality

The AI that processes medical data must comply with:

- *The General Data Protection Regulation (GDPR)—Regulation (EU) 2016/679, particularly Art. 9 (sensitive data) and Art. 22* (automated decisions with a significant impact on individuals; Government of Romania, 2000).
  Regulation (EU) 2016/679 Of The European Parliament and of the Council of 27 April 2016 on the protection of natural persons about the processing of personal data and on the free movement of such data, and repealing Directive 95/46/EC [General Data Protection Regulation, European Parliament, for EU laws, 2016].^3^
- Law no. 190/2018 regarding the measures for the implementation of GDPR in Romania, which sets specific rules for processing health data. Law No. 190/2018 on implementing measures for Regulation (EU) 2016/679 of the European Parliament and of the Council of 27 April 2016 on the protection of natural persons about the processing of personal data and on the free movement of such data, and repealing Directive 95/46/EC (General Data Protection Regulation; Law no. 190 of 18 July 2018, on measures implementing regulation (EU) 2016/679 of the European Parliament and of the council of 27 April 2016 on the protection of natural persons about the processing of personal data and on the free movement of such data, and repealing directive 95/46/EC (general data protection regulation), issuer, parliament of Romania, published in the official gazette no. 651 of 26 July 2018, 2018)

Non-compliance with these norms may be sanctioned by the National Supervisory Authority for Personal Data Processing (ANSPDCP; Law no. 190 of 18 July 2018, on measures implementing regulation (EU) 2016/679 of the European Parliament and of the council of 27 April 2016 on the protection of natural persons about the processing of personal data and on the free movement of such data, and repealing directive 95/46/EC (general data protection regulation), issuer, parliament of Romania, published in the official gazette no. 651 of 26 July 2018, 2018); National Supervisory Authority - Government of Romania, 2018).

##### The directive regarding liability for defective products

- *EU Directive No. 2024/2853* on liability for defective products and repealing Directive No. 85/374 (the “*LPD Directive*”). This Directive is to apply to products placed on the market or put into service after 9 December 2026. Member States must bring into force the legislative acts transposing this Directive by 9 December 2026 [Directive (EU) 2024/2853 of the European Parliament and of the council of 23 October 2024 on liability for defective products, 2024].
- *Directive 85/374/EEC* (European Economic Community; *Council Directive 85/374/EEC of 25 July 1985 on the approximation of the laws, regulations and administrative provisions of the Member States concerning liability for defective products)* regarding the liability for defective products (revised in 2024) includes medical software and AI in the category of the products for which the manufacturer can be held directly liable (Council Directive 85/374/EEC of 25 July 1985 on the approximation of the laws, regulations and administrative provisions of the member states concerning liability for defective products, n.d.).

This imposes the objective liability of the manufacturer for the harm caused by product defects, irrespective of fault.

#### Who is liable in case harm is caused by medical AI?

In the event of harm resulting from the use of AI in medicine, liability may be attributed to several entities:

##### The liability of the doctor

- *Art. 643 of Law no. 95/2006* stipulates that doctors are liable for the harm caused to patients in the case of a professional error (Law no. 95/2006 on the reform in the field of healthcare, n.d.).
- If AI is used only as a decision support tool and the doctor makes the final decision, the doctor may be held liable if it is proven that he/she inappropriately used AI.

*Example:* A doctor relies exclusively on an AI-generated recommendation to diagnose a patient, without checking the correctness of the diagnosis, and the patient is harmed. In this case, the doctor may be held liable for malpractice.

##### The liability of the healthcare facility

- The hospitals and the clinics may be liable under *Art. 1,357 of the Civil Code and Art. 644 of Law no. 95/2006* if negligence in the selection, implementation, or supervision of an AI system leads to harm to the patient (Law no. 95/2006 on the reform in the field of healthcare, n.d.; Civil Code, n.d.).
- If the medical facility uses AI as an autonomous decision (without the doctor’s involvement), it can be directly liable.

Example: A hospital implements an AI system for patient triage, but the algorithm has a bias that causes underdiagnosis of certain categories of patients. The hospital may be liable for the harm done.

##### The liability of the software manufacturer

- According to the *MDR Regulation and the Directive regarding the liability for defective products*, the manufacturer of an AI software may be liable if its defects cause harm [Regulation (EU) 2017/745 on medical devices (MDR), n.d.; Directive (EU) 2024/2853 of the European Parliament and of the council of 23 October 2024 on liability for defective products, 2024; Regulation (EU) 2017/746 of the European Parliament and of the council of 5 April 2017 on in vitro diagnostic medical devices, 2017; Regulation (EU) 2023/988 of the European Parliament and of the Council of 10 May 2023 on general product Safety, n.d.].
- If the AI is trained on a faulty data set and produces systematically erroneous decisions, the manufacturer may be liable for a defective product [Regulation (EU) 2017/745 on medical devices (MDR), n.d.; Directive (EU) 2024/2853 of the European Parliament and of the council of 23 October 2024 on liability for defective products, 2024; Regulation (EU) 2017/746 of the European Parliament and of the council of 5 April 2017 on in vitro diagnostic medical devices, 2017; Regulation (EU) 2023/988 of the European Parliament and of the Council of 10 May 2023 on general product Safety, 2023].

Example: An AI algorithm for diagnosing skin cancer was trained predominantly on images of Caucasian patients and misses the diagnosis of patients with darker skin. If a patient is harmed, the software manufacturer may be liable.

#### Relevant current and future regulations

##### The artificial intelligence act (AI act)

- The European Commission’s 2021 proposal for the AI Regulation (AI Act) and adopted in 2024 (*Artificial Intelligence Act, 21 May 2024)* classifies the AI used in medicine as a “high-risk AI system” [Regulation laying down harmonised rules on artificial intelligence (artificial intelligence act), 2024].

It imposes strict requirements for *transparency, auditability, human intervention, and safety,* which will influence both users (doctors, hospitals) and software developers.

##### Revision of the directive regarding liability for defective products

- EU Directive No. 2024/2853 (*LPD* Directive) on liability for defective products and repealing Directive No. 85/374/EEC. The 2022 proposal extends liability to the software manufacturers, including AI, and eliminates the necessity for the victims to prove a specific algorithmic defect to obtain compensation. After damages [Directive (EU) 2024/2853 of the European Parliament and of the council of 23 October 2024 on liability for defective products, 2024].

#### Conclusions and recommendations

- Currently, liability for the use of AI in medicine is assessed according to the general regulations regarding medical malpractice, consumer protection, and medical devices.
- The AI Act and the new rules regarding product liability will bring more clarity and legal responsibility for AI users and developers.
- Doctors and medical facilities must implement clear protocols for the use of AI, avoid exclusive dependence on AI in decision-making, and observe the principles of medical ethics and data protection.

AI can help doctors make the best decisions. Science must progress for the benefit of people, of course, within an appropriate legal framework. AI is revolutionizing healthcare, but it may create new liabilities for manufacturers and users alike. AI is already being used for the diagnosis and treatment of cancer, with AI-equipped surgical robots becoming more and more present in its treatment (prostatectomies, etc.; see Footnote 1).

Naturally, as the use of AI increases, so do the risks and the liability associated with it. In order not to reduce the use and to maximize the application of the AI system, the risks and the liability must be very well defined, so that all the parties involved (patient, doctor, device) understand their responsibilities and the implicit legal liability when the technology inevitably causes harm. Current and future medical insurance must be prepared for new types of malpractice at a pace which should keep up with the pace of AI system development (France needs a public label for the evaluation of AI used in healthcare, 2024; Carter et al., 2020).

Essential elements of patient care (especially of the oncology patient), such as clinical examination, compassion, clinical intuition, and empathy, make medicine a distinctive science and will probably determine predominantly human medical care, in many cases, which will exceed AI-dominated care. However, in the future, AI will play an increasingly major role in medical decision-making. AI influences medical malpractice liability by adding new causes and the appearance of new damages from this point of view, a judge will have to rule on the new malpractice cases very carefully and determine whose fault it is: the doctor’s, the AI manufacturers’, and/or the hospital’s (L’IA dans le domaine de la santé: un immense potentiel, d’énormes risques, 2024; Price et al., 2022; Hodge, 2022; Laptev et al., 2022; Mittelstadt, 2021; Kamensky, 2020; Lupton, 2018; Jorstad, 2020; Król-Całkowska & Walczak, 2021).

A doctor’s decision to follow or not follow AI predictions and to use the system will have legal consequences. New issues will arise, such as doctors who are trained to work with AI technology (e.g., AI-assisted robotic surgery) but who never learned to operate without it, will have difficulties when computers fail, which could lead to malpractice liability issues if the doctor is not prepared to complete an operation or solve a complication, for example, without the robot. Cause-and-effect relationships will play an important role, because it will be difficult for a judge to analyze whose responsibility it is when the doctor’s responsibilities are shared with AI systems. Here, the opinion of an expert in the field will likely be required (Gonçalves, 2018; Jobson et al., 2022; Smith & Fotheringham, 2020; Tripathi et al., 2024; Shah et al., 2020; Parikh et al., 2019).

## Conclusion

Doctors must be the human interface between technology and the patient by responsibly using AI in the diagnosis and treatment of a disease, especially neoplastic diseases, so that essential elements such as compassion and empathy do not disappear from medical practice. As we have sought to emphasize in this paper, we must prepare the legal framework to provide the parties involved (doctor, technology manufacturer, hospital) with a reasonable capacity for the development and application of AI and ML systems, to anticipate liability issues from a legal point of view, so that these technologies may continue to develop rapidly and revolutionize medical care.

## Data Availability

The datasets presented in this study can be found in online repositories. The names of the repository/repositories and accession number(s) can be found at: virgiliuprunoiu@yahoo.com.

## Footnotes

1 https://www.gustaveroussy.fr/en/france-set-become-global-leader-using-ai-diagnose-and-treat-diseases. (Accessed 18.02.2026).

2 https://www.capital.ro/regulamentul-ia-inteligenta-artificiala-uniunea-europeana.html. (Accessed 13.08.2024).

3
https://gdpr-info.eu/
